# Bioactive Potential of the Wild Edible Fungus *Rhizopogon roseolus* (Corda) Th. Fr.

**DOI:** 10.3390/metabo16030176

**Published:** 2026-03-06

**Authors:** Elif Yürümez Canpolat

**Affiliations:** Department of Biotechnology, Faculty of Science, Niğde Ömer Halisdemir University, 51240 Niğde, Türkiye; elif.yurumez@ohu.edu.tr

**Keywords:** *Rhizopogon roseolus*, bioactivity, phenolic compounds, antioxidants, fatty acids, antimicrobial activity

## Abstract

**Backgrouınd/Objectives:** Edible fungi are increasingly regarded as important natural reservoirs of secondary metabolites exhibiting a wide range of biological activities. The present study aimed to molecularly identify *Rhizopogon roseolus* collected from Türkiye and to systematically evaluate its bioactive properties. **Methods:** The antimicrobial and antioxidant activities of methanolic, ethanolic, and aqueous extracts were evaluated. The phenolic profile and the fatty acid composition of the species were characterized using HPLC-DAD and GC–MS respectively. **Results:** All extracts showed noticeable antimicrobial activity against several pathogenic microorganisms, including *Bacillus subtilis*, *Micrococcus luteus*, *Bacillus cereus* and *Pseudomonas aeruginosa*. In addition, the extracts displayed remarkable antioxidant potential. The methanolic extract of *R. roseolus* demonstrated DPPH and ABTS radical scavenging activities of 651.44 ± 15.02 mg TE/g and 162.71 ± 8.11 mg TE/g, respectively. Its ferric reducing antioxidant power was determined as 724.33 ± 12.23 mg AAE/g DW, while the cupric reducing antioxidant capacity reached 952.45 ± 4.35 mg TE/g DW. These strong antioxidant effects were closely associated with the high phenolic content (9.89 ± 0.08 mg GAE/g DW) of the extracts. GC–MS analysis indicated that palmitoleic, oleic, and linoleic acids were the major fatty acids, while HPLC-DAD revealed that gallic acid and pyrocatechol were major phenolic compounds present in *R. roseolus*, suggesting a nutritionally beneficial metabolite composition. **Conclusions:** Taken together, the findings demonstrate that *R. roseolus* represents a promising natural source of antimicrobial and antioxidant compounds with potential applications.

## 1. Introduction

Mushrooms have long been appreciated both as food and as components of traditional medicine. Many edible macrofungi are characterized by low caloric content while being rich sources of vitamins, essential amino acids, and minerals [1]. The genus Rhizopogon comprises so-called “false truffle” fungi that establish ectomycorrhizal associations with trees of the Pinaceae family, particularly pine species. Among them, *Rhizopogon roseolus* is a widely distributed species that was first described in Europe in the nineteenth century [2].

This species typically grows just below or directly on the soil surface in pine forests and has been used as a model organism in afforestation programs, as its mycelium enhances seedling development through symbiotic interactions with tree roots. *R. roseolus* is also a naturally occurring macrofungus in Türkiye, especially in regions dominated by pine forests [3,4]. Owing to its edibility, this mushroom is collected and consumed by local populations. In Japan, it is known as “shōro” and is regarded as a highly prized and flavorful truffle [5].

The medicinal potential of mushrooms is largely attributed to their rich content of bioactive compounds. Many mushroom species contain a wide range of biologically active constituents, including phenolics, terpenes, sterols, and polysaccharides. These compounds are responsible for various biological effects, such as antifungal, antioxidant, antiproliferative, anticancer, and immunomodulatory activities [6]. Numerous studies have investigated the biochemical composition and biological properties of *R. roseolus*, reporting variations that may be associated with ecological conditions or differences in extraction and analytical methodologies [7,8,9,10]. Collectively, these findings indicate that *R. roseolus* is a species of considerable interest not only for its nutritional value but also for its pharmacological potential. Antioxidant and antimicrobial activities are among the most intensively investigated topics in contemporary biomedical research. Excessive accumulation of free radicals in the body contributes to the onset and progression of numerous chronic disorders, including cancer, atherosclerosis, diabetes, and various degenerative diseases. Although endogenous antioxidant defense systems exist, factors such as aging, environmental stressors, and infections can impair this balance, resulting in oxidative damage. For this reason, increasing attention has been directed toward the health benefits of natural antioxidants obtained through diet or supplementation. In this context, mushrooms have attracted considerable interest owing to their pronounced antioxidant potential [7,11]. In parallel, the global rise in antibiotic resistance and the increasing prevalence of infectious diseases have intensified the search for novel antimicrobial agents derived from natural sources. Fungi represent a particularly promising reservoir of such compounds, as they produce a wide array of bioactive metabolites. Fungal sterols, especially ergosterol, as well as diverse phenolic compounds and terpenoids, have been reported to exhibit significant antimicrobial and immunomodulatory activities, highlighting their potential role in the prevention and control of infectious diseases [12,13,14].

In this study, *R. roseolus* collected from the Niğde province of Türkiye was subjected to a comprehensive biochemical investigation. The study aimed to evaluate total phenolic content, antioxidant capacity, fatty acid composition, and antimicrobial activity. Methanolic, ethanolic, and aqueous extracts were examined for their free radical scavenging activities using DPPH and ABTS assays, as well as for their reducing power through FRAP and CUPRAC methods. The phenolic composition of the extracts was further characterized by HPLC-DAD analysis. The fatty acid profile of *R. roseolus* was determined by GC–MS analysis, while the antimicrobial activity of the extracts was assessed against selected Gram-positive and Gram-negative bacterial strains using the disk diffusion method. The findings of this study provide detailed biochemical insights into *R. roseolus* and support its potential applicability in nutraceutical and pharmaceutical contexts. Furthermore, to the best of our knowledge, this work represents the first comprehensive biochemical characterization of *R. roseolus* collected from Türkiye.

## 2. Materials and Methods

### 2.1. Material

Fungal samples were collected in October 2021 from the campus area of Niğde Ömer Halisdemir University (37.943° N, 34.622° E). The samples were photographed in their natural habitat, and information about the environmental conditions, along with the morphological structures, was noted. Fungal samples were identified due to morphological characteristics such as color, shape, and size of basidiocarp, and spores based on the current literature [15,16,17,18]. The samples were air-dried and stored under laboratory conditions for further use. A voucher specimen (Voucher No: NOHU4805) was deposited in the Herbarium of Niğde Ömer Halisdemir University.

### 2.2. Molecular Identification

By comparing the sequence data with molecular databases, molecular techniques such as isolation, amplification, and sequencing of the conserved fragments of nuclear DNA across the evolutionary stage enable a more precise identification of mushroom samples. The Internal Transcribed Spacer (ITS) region of the rDNA, which is around 700 bp long and a highly conserved segment, is mostly employed for fungus species identification due to its significant inter-species variation [19,20].

DNA extraction from samples was performed using the Nucleospin Plant II DNA extraction kit (Macherey-Nagel, Düren, Germany) according to the manufacturer’s instructions with some minor modifications. The ITS fragment of rDNA was amplified by polymerase chain reaction (PCR) using the universal ITS-1 (5′-TCCGTAGGTGAACCTGCGG-3′) and ITS-4 (5′-TCCTCCGCTTATTGATATGC-3′) primers [21]. PCR amplification was conducted using ITS1 and ITS4 primers in a 50 µL reaction mixture containing 10 mM forward and reverse primers, Dream Taq Master mix (Thermo Fisher Scientific Inc., Waltham, MA, USA), the DNA template and nuclease free water. Reaction conditions consisted of preheating at 94 °C for 2 min and the subsequent 30 cycles of 95 °C (30 s), 60 °C (30 s), and 72 °C (1 min). After the completion of 30 cycles, final chain elongation was conducted at 72 °C for 5 min. Following the gel electrophoresis, the PCR product was sent for sequence analysis (BM Labosis, Ankara, Türkiye). The sequence data of the sample were compared with the NCBI GenBank database via nucleotide BLAST for identification of the macrofungus.

### 2.3. Extract Preparation

Dried macrofungus sample was powdered by a commercial blender. Two grams of powdered material was mixed with 50 mL of solvent (methanol, ethanol, distilled water); and homogenized for 3 min at 7500 rpm using a HG-15D homogenizer (Daihan Scientific, Gangwon-do, Korea). The use of solvents with different polarities (methanol, ethanol, and distilled water) was intended to ensure the extraction of a wide spectrum of bioactive compounds with varying chemical properties. The mixture was then incubated overnight at ambient temperature in the dark followed by ultrasonication at 35 kHz for 45 min at 30 °C using an ultrasonic bath (Sonorex, Bandelin, Berlin, Germany). The extract was filtered through Whatman No. 1 filter papers, and the solvent was removed by a rotary evaporator (HeiVap Value, Heidolph, Schwabach, Germany). The residue was recovered with enough extraction solvent or 5% DMSO for obtaining 100 mg/mL extract and these extracts were used for the antioxidant and antimicrobial activity assays, respectively. The extractions were performed as three replicates and the extracts were kept at 4 °C for further analysis [22]. The extraction yield was calculated using the following formula;

W_2_ − W_1_/W_0_ × 100,

where W_2_ is the weight of the extract and the container, W_1_ the weight of the container alone and W_0_ the weight of the initial dried sample.

### 2.4. Total Phenolic Compounds

The total content of phenolic compounds was quantified using Folin–Ciocalteu’s phenol reagent method [23]. Briefly, 100 µL of extract was mixed with a tenfold diluted 2N Folin–Ciocalteu’s phenol reagent and incubated for 5 min. Then, 7.5% NaCO_3_ solution was added to the mixture and incubated for 90 min at ambient temperature in the dark. The bluish color formed in the test tube was measured at 765 nm using a spectrophotometer. The measurements were conducted in triplicate, and the results were expressed as mean ± standard deviation in mg GAE/g DW.

### 2.5. Determination of Phenolic Composition

The phenolic profiles of *R. roseolus* extracts were investigated using a Shimadzu Nexera 2 HPLC-DAD system (Kyoto, Japan) equipped with a reversed-phase ODS C18 column (4.6 × 250 mm, 5 μm particle size; GL Science, Tokyo, Japan) maintained at 30 °C. Separation of phenolic constituents from 20 µL sample was achieved by gradient elution with solvent A (2% acetic acid in distilled water) and solvent B (methanol) at a constant flow rate of 1.0 mL/min. The gradient program was as follows: 95% A/5% B at 0 min; 80% A/20% B at 18 min; 60% A/40% B at 30 min; 50% A/50% B at 40 min; 100% A at 50 min; and re-equilibration to 95% A/5% B at 57 min. Chromatographic detection was performed at 280 nm, as most phenolic compounds exhibit strong absorbance at this wavelength. Identification of individual phenolic compounds was based on comparison of their retention times with those of authentic reference standards [24].

### 2.6. Antioxidant Activity

#### 2.6.1. DPPH Assay

2,2-diphenyl-1-picrylhydrazyl (DPPH) comprising stable free radicals, has the maximum light absorbance at 517 nm. The ability of the fungal extracts to scavenge the DPPH solution indicates antioxidant capability. The DPPH radical scavenging activity assay was performed by a modified method originally reported by Blois [25,26]. DPPH scavenging values of Trolox solutions with various concentrations (100–2000 µM) were used to establish a standard curve, and the results were presented as mean ± standard deviation in mg TE/g DW.

#### 2.6.2. ABTS Assay

The inhibition effect of samples on ABTS^+^ is another widely used method for determining the antioxidant capacity. The scavenging capability of the macrofungus extract against 2-azino-bis-(3-ethylbenzothiozoline-6-sulphonic acid) (ABTS) solution is the indicator of the antioxidant capacity. The ABTS radical scavenging activity assay was performed according to the method reported by Mohtar et al. [27]. The standard curve was established using the inhibitory values of Trolox solutions at different concentrations (20–1000 µM), and the results were presented as mean ± standard deviation in mg TE/g DW.

#### 2.6.3. FRAP Assay

The antioxidative activity of the sample, which reacts with potassium ferricyanide (K_3_[Fe(CN)_6_]) to create potassium ferrocyanide (K_4_[Fe(CN)_6_]), might be associated with an increase in the absorbance of ferric ferrocyanide, a blue-colored complex with a maximum absorbance at 700 nm. With a few minor adjustments, the FRAP assay was carried out in accordance with existing literature [28]. The data were evaluated using the ascorbic acid standard curve (1–100 mM), and the findings were expressed as mean ± standard deviation in mg AAE/g DW.

#### 2.6.4. CUPRAC Assay

In cases where substances functioning as electron donors are present, the color of the neocuproine and Cu(II)Cl_2_ mixture changes from bright yellow to orange, indicating the antioxidant ability of a sample by converting cupric (Cu^+2^) ions to cuprous (Cu^+^) ions. With a few minor adjustments, the CUPRAC assay was carried out using the protocol first described by Apak et al. [29]. The absorbance of samples was measured at 450 nm against a blank solution. The antioxidant capacity of mushroom samples with varying concentrations was assessed using the Trolox standard curve (20–1000 µM), and the results were presented as mean ± standard deviation in mg TE/g DW.

#### 2.6.5. Determination of Fatty Acid Composition

The fatty acid composition of the macrofungus sample was determined by a Shimadzu QP2010 Ultra (Kyoto, Japan) gas chromatography–mass spectrometry (GC-MS) system with a Restek Rxi-5MS (Restek Corporation, Bellefonte, PA, USA) column as reported in a previous study with minor changes [30]. Two g of sample was used for extraction; thus, the solvent volume was increased proportionally. The analyses were performed in the Central Research Laboratory of Niğde Ömer Halisdemir University (Niğde, Türkiye).

### 2.7. Antimicrobial Activity

The antimicrobial activity of *R. roseolus* extracts was evaluated by the disk diffusion method. The tested microorganisms included a total of 21 strains, consisting of 5 food-related isolates (FI) (*Enterococcus faecium*, *Salmonella kentucy*, *Salmonella infantis*, *Klebsiella pneumoniae*, *Listeria innocua*) and 4 clinical isolates (CI) (*Streptococcus mutans*, *Klebsiella pneumoniae*, *Candida tropicalis*, *Staphylococcus aureus (MRSA)*), together with 12 reference strains: *Bacillus cereus* RSKK 863, *B. subtilis* DSMZ 1971, *Enterobacter aerogenes* ATCC 13048, *Escherichia coli* ATCC 25922, *Micrococcus luteus* M41, *Proteus vulgaris* ATCC 13315, *Pseudomonas aeruginosa* DSMZ 50071, *Salmonella typhimurium* SL1344, *Staphylococcus aureus* ATCC 25923, *S. epidermidis* DSMZ 20044, *Shigella flexneri* RSKK 184 and the yeast *Candida albicans* ATCC 10231 which were routinely grown in Luria–Bertani (LB) broth medium. Microbial inoculate were adjusted to 0.5 McFarland standard and uniformly spread onto LB plates using a sterile Drigalski spatula under aseptic conditions. The extracts obtained for the antimicrobial activity assay were dissolved in distilled water containing 5% DMSO. Sterile paper disks with a diameter of 6 mm, containing 30 µL extract, were placed on the agar media and incubated for 24 h at 37 °C. Following the incubation, the diameters of inhibition zones formed around the disks were measured. In this study, sterile blank disks and disks loaded with ethyl alcohol, methanol, and distilled water were used as negative controls. As positive controls, Ciprofloxacin (CIP) and Tobramycin (TOB) for strains were utilized to enable comparison of the results obtained. All experiments were conducted in triplicate, and the inhibition zone diameters were expressed as mean ± standard deviation (mm) [31].

### 2.8. Statistical Analysis

The data presented in this study were analyzed using IBM Statistical Package for Social Sciences software (SPSS ver. 24.0, IBM, Chicago IL, USA). The one-way analysis of variance (ANOVA) was performed to compare the means among the groups at a significance level of *p* < 0.05, followed by Tukey’s post hoc test.

## 3. Results

### 3.1. Identification of Macrofungus

**Figure 1:**
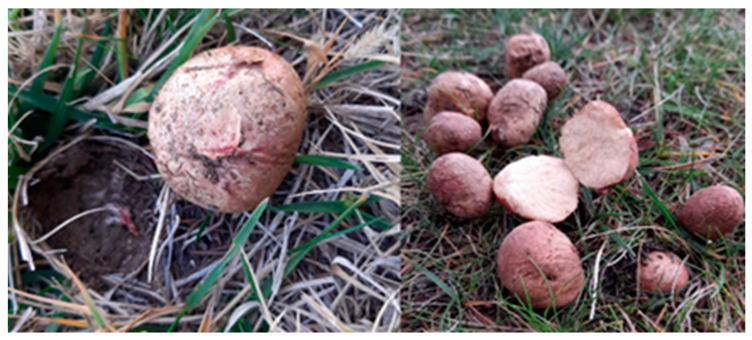
The field images of *R. roseolus* collected from Niğde, Türkiye.

The macrofungus sample that constitutes the material of this study was collected under a *Pinus* sp. in meadow from a pine forest located in Niğde Ömer Halisdemir University (37.943° N, 34.622° E, altitude: 1270 m) and identified due to its morphological characteristics (Figure 1) according to the current literature [15,16,17,18]. Additionally, identification using molecular techniques based on the amplification and sequence analysis of the ITS fragment of the rDNA was also performed. The obtained sequence was compared with reference sequences in the GenBank database using BLAST analysis and showed 99.2% similarity with *Rhizopogon roseolus*, confirming the molecular identity of the specimen. The sequence has been deposited in GenBank under Accession Number PX441692. According to both morphological and molecular identification analysis, the result revealed that the macrofungus sample was *Rhizopogon roseolus* (Corda) Th. Fr. (1909).

### 3.2. Antioxidant Activity

*R. roseolus* extracts were analyzed in terms of antioxidant capability via various methods based on free radical (DPPH and ABTS) scavenging activity and metal ion reducing (FRAP and CUPRAC) capacity assays, along with the determination of total content of phenolic compounds via Folin–Ciocalteu’s phenol reagent method. The results are given in Table 1.

Table 1 reveals that the methanol extract of *R. roseolus* exhibited high free radical scavenging activity with 651.44 ± 15.02 mg TE/g DW DPPH and 162.71 ± 8.11 mg TE/g DW ABTS scavenging values. Moreover, with 724.33 ± 12.23 mg AAE/g DW and 952.45 ± 4.35 mg TE/g DW values *R. roseolus* methanol extract showed high iron (FRAP) and copper (CUPRAC) ion reducing capacities, respectively. It was also indicated that *R. roseolus* methanol extract contained 9.89 ± 0.08 mg GAE/g DW phenolic compounds.

The present study demonstrated the phenolic profiles of *R. roseolus* extracts, as analyzed through HPLC. Methanol extracts were found to possess higher concentrations of both phenolic acids and flavonoids compared to aqueous and ethanol extracts. Among the identified phenolic compounds, pyrocatechol, known for its potent antioxidant properties, was detected at the highest concentration (169.41 µg/g), followed by gallic acid (88.83 µg/g) and catechin (73.24 µg/g) in the methanolic extract of *R. roseolus* (Table 2). Moderate amounts of epicatechin (33.82 µg/g) and caffeic acid (17.12 µg/g) were also observed, whereas p-coumaric acid (4.91 µg/g) and quercetin (5.13 µg/g) were present at comparatively lower levels.

**Figure 2:**
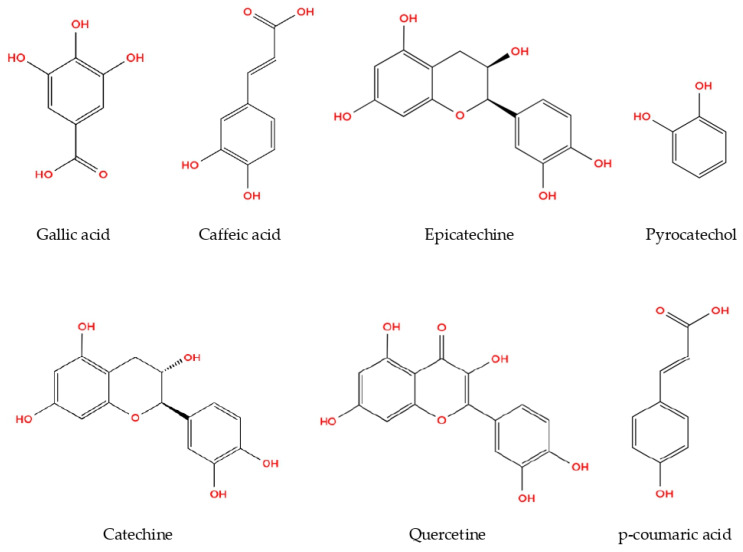
Phenolic compounds from *R. roseolus* methanol extract determined by HPLC-DAD.

The phenolic concentrations detected in *Rhizopogon* extracts fall within the ranges previously reported for edible macrofungi. Gallic acid and pyrocatechol were the dominant phenolics, whereas flavonoids such as catechin, epicatechin, and quercetin were present at moderate levels. The relatively high pyrocatechol content suggests that Rhizopogon may represent a phenolic-rich fungal resource. The chemical structures of the identified phenolic compounds are depicted in Figure 2.

On the other hand, the ethanol extract of *R. roeolus* with a 8.97 ± 0.07 mg GAE/g DW phenolic content exhibited free radical scavenging activities against DPPH and ABTS with 601.01 ± 10.18 mg TE/g DW and 157.68 ± 7.08 mg TE/g DW, respectively. The ethanol extract also showed 711.52 ± 3.74 mg AAE/g DW FRAP and 912.49 ± 4.34 mg TE/g DW CUPRAC values.

**Figure 3:**
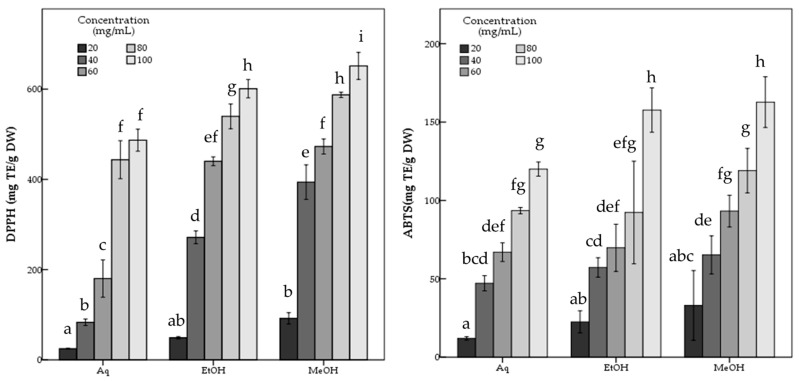
Free radical scavenging activity of *R. roseolus* extracts at various concentrations. DPPH radical scavenging activity and ABTS^+^ scavenging activity (mg TE/g DW). Different lowercase letters indicate a significant difference at *p* < 0.05.

**Figure 4:**
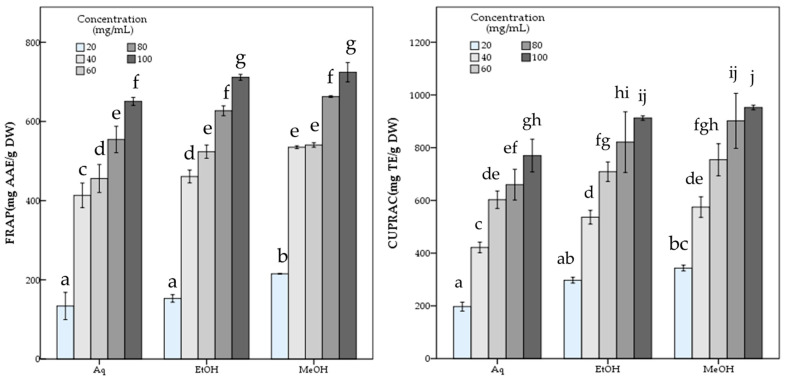
Metal ion reducing capacity of *R. roseolus* extracts at various concentrations. Ferric reducing antioxidant power (FRAP) (mg AAE/g DW) and Cupric reducing antioxidant capacity (CUPRAC) (mg TE/g DW). Different lowercase letters indicate a significant difference at *p* < 0.05.

The aqueous extract showed significantly (*p* < 0.05) lower antioxidant potential than both methanol and ethanol extracts with 486.81 ± 12.21 mg TE/g DW DPPH, and 119.98 ± 2.26 mg TE/g DW ABTS scavenging activity and 650.72 ± 5.11 mg AAE/g DW FRAP and 770.11 ± 31.02 mg TE/g DW CUPRAC capacity values, while containing 7.90 ± 0.22 mg GAE/g DW phenolic compounds. The statistical analysis indicated the significant differences among the *R. roseolus* extracts at *p* < 0.05 in TPC, DPPH, ABTS, FRAP and CUPRAC values (Table 1). Moreover, Figure 3 and Figure 4 demonstrate the antioxidant activity trend of *R. roseolus* extracts at varying concentrations.

### 3.3. Determination of Fatty Acid Composition

The fatty acid composition of *R. roseolus* was determined by GC-MS, and the results are given in Table 3.

According to Table 3 it was indicated that the majority of fatty acid composition of *R. roseolus* was composed of unsaturated fatty acids including methyl esters of oleic acid (35.37%, RT: 41.929), linoleic acid (25.03%, RT: 41.809), palmitoleic acid (3.33%, RT:37.842), and 8,11,14-Docosatrienoic acid (0.29%, RT:45.678), along with the ethyl esters of oleic acid (8.23%, RT:43.252), linoleic acid (6.35%, RT:43.147), and palmitoleic acid (0.89%, RT:39.4).

**Figure 5:**
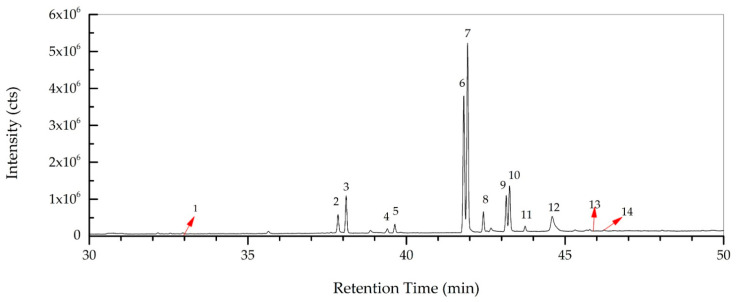
Total ion chromatogram (TIC) of *R. roseolus* fatty acids determined by GC-MS.

**Figure 6:**
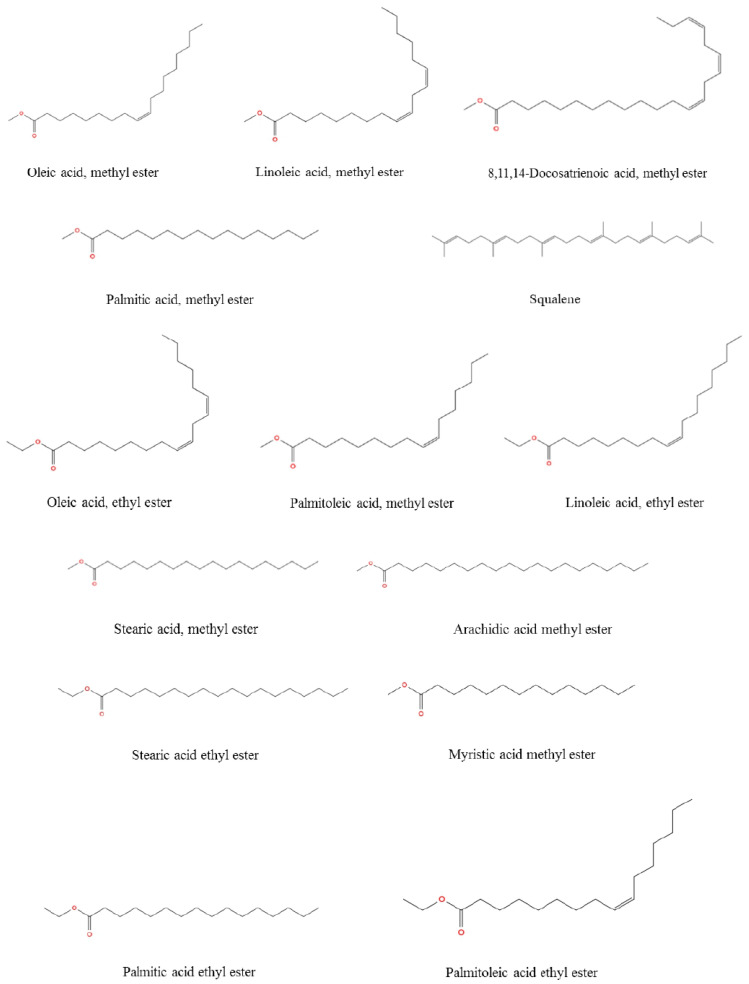
The chemical structure of the fatty acids from *R. roseolus* determined by GC-MS.

The total concentration of mono- and polyunsaturated fatty acids were found to be 79.49%. On the other hand, methyl esters of palmitic acid (6.42%, RT: 38.098) and stearic acid (3.44%, RT: 42.429) were the major saturated fatty acids determined in *R. roseolus*, along with arachidic acid (0.20%, RT: 46.231) and myristic acid (0.19%, RT: 32.95), and ethyl esters of palmitic acid (1.46%, RT: 39.634) and stearic acid (0.89%, RT: 43.743). The total concentration of saturated fatty acids was determined to be 12.6%. Squalene, which is a precursor triterpenoid that takes part in sterol biosynthesis, was also determined with a concentration of 6.25%. The total concentration of the identified compounds was determined as 98.34% from the fatty acid composition analysis performed by GC-MS. The total ion chromatogram (TIC) and the chemical structures of the determined fatty acids are depicted in Figure 5 and Figure 6.

### 3.4. Antimicrobial Activity

The antimicrobial activity of *R. roseolus* methanol, ethanol and aqueous extracts was tested against some Gram-positive (+) and Gram-negative (–) bacterial strains, as well as yeast strains *Candida albicans* and *Candida tropicalis* using the disk diffusion method.

**Figure 7:**
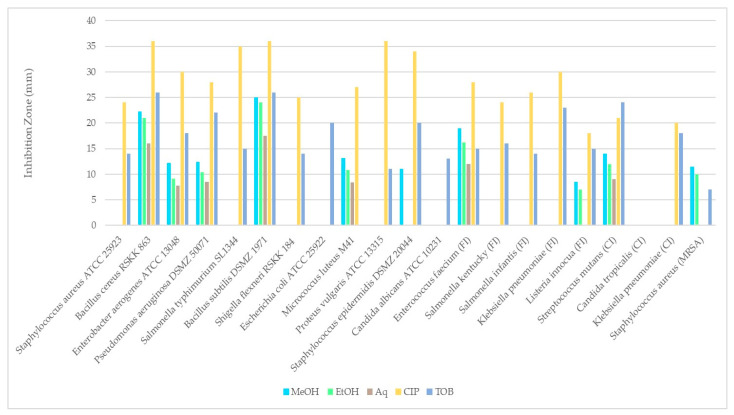
Comparison of the disk diffusion test results of *R. roseolus* extracts against test microorganisms.

Table 4 and Figure 7 shows the antimicrobial activities of extracts obtained from *R. roseolus*. The obtained results revealed that the methanol extract of *R. roseolus* exhibited the highest antimicrobial activity against *B. subtilis* with an inhibition zone diameter of 25.0 ± 0.35 mm, followed by *B. cereus* with 22.25 ± 0.35 mm and *E. faecium* with 19.00 ± 0.71 mm. The methanol extract also demonstrated antimicrobial activity against *S. mutans*, *M. luteus* M41, and *S. aureus* (MRSA), with inhibition zones of 14.00, 13.13 and 11.50 mm, respectively. No inhibitory effect was observed against *S. aureus*, *S. typhimurium*, *K. pneumoniae*, *S.kentucky*, *S. infantis*, *E. coli*, *P. vulgaris*, *S. flexneri* and *C. albicans*, *C. tropicalis* strains. The largest inhibition zone was determined as 24.00 ± 0.71 mm against *B. subtilis* for ethanol extract, followed by *B. cereus* with 21.00 ± 0.71 mm and *E. faecium* with 16.25 ± 1.41 mm. The largest inhibition zone for the aqueous extract was observed against *B. subtilis* with 17.50 ± 1.06 mm followed by *B. cereus* and *E. faecium* with 16.00 ± 1.41 and 12.00 ± 1.06 mm, respectively.

These findings indicate that methanol is an effective solvent for extracting bioactive compounds from the fruiting bodies of *R. roseolus*. All extracts exhibited low to high antimicrobial activity against several tested Gram-positive and Gram-negative microorganisms, including both foodborne and clinical strains. The results revealed that *Bacillus* species were more sensitive than the other tested microorganisms, showing the highest antibacterial activity among all.

## 4. Discussion

This study focused on the determination of antioxidant potential and antimicrobial activity of *R. roseolus* (Chorda) Th Fr collected from Niğde (Türkiye), following the molecular identification. The extracts of the macrofungus sample were obtained by three different solvents, ethanol, methanol and distilled water. The fatty acid profile was also determined by GC-MS. In addition, individual phenolic compounds were identified and quantified by HPLC-DAD analysis. The total phenolic content and antioxidant activity values obtained in the present study clearly indicate that *R. roseolus* possesses a pronounced antioxidant capacity. These findings are in good agreement with previous reports on *Rhizopogon* species, supporting the view that members of this genus represent valuable sources of natural antioxidants [32,33].

Gursoy et al. (2010) [32] reported that *R. roseolus* exhibited lower phenolic content when compared with certain other wild mushrooms, such as *Ramaria flava*, which displayed the highest phenolic concentration among the species analyzed in their study. Nevertheless, the phenolic levels detected in *R. roseolus* remain comparable to those reported for other edible fungi and the antioxidant activity measured in this study supports the contribution of these phenolic compounds to the overall antioxidant capacity. The high antioxidant activity values obtained in this study may be attributed to the phenolic profile and other secondary metabolites of *R. roseolus*. Although antioxidant activity is often correlated with total phenolic content, the type and potency of individual phenolic compounds are also highly important. While the total phenolic content of *R. roseolus* extract was not as high as that of *R. flava*, specific phenolic acids or flavonoids it contains may exert particularly strong antioxidant effects [32].

A study investigating the phenolic compounds, volatile oils, metal content, antioxidant, and antibacterial activities of *Rhizopogon luteolus*, *Russula delica*, *Marasmius oreades*, *Clitocybe geotropa* and *Ramaria aurea* indicated that *R. luteolus* (RL) possessed a notably high total phenolic content (2.31 ± 0.10 mg GAE/g dry weight) compared to the other tested fungi. Phenolic compounds are widely recognized as major contributors to the antioxidant potential of mushrooms due to their capacity to scavenge free radicals and inhibit oxidative processes. In this context, the strong total antioxidant activity exhibited by RL—exceeding that of standard antioxidants such as α-tocopherol—can be largely attributed to its rich phenolic composition. Similar observations have been reported for other phenolic-rich wild edible mushrooms, where a positive correlation between total phenolic content and antioxidant activity has been consistently demonstrated. Moreover, environmental factors, habitat conditions, and species-specific metabolic pathways are known to influence phenolic biosynthesis in mushrooms, which may explain the comparatively high antioxidant performance of RL. The high phenolic content and antioxidant capacity of RL suggest a multifunctional bioactive profile, supporting its potential application as a natural source of antioxidant and antimicrobial agents [34]. These findings reinforce the growing interest in *Rhizopogon* species as promising candidates for functional food development and nutraceutical applications, particularly in the context of oxidative stress-related disorders.

This study indicated that *R. roseolus* collected from Niğde exhibited higher antioxidant capability and contained higher amounts of phenolic compounds than the samples collected from other regions of Türkiye [32,33,34,35]. This regional variation in bioactivity may be attributed to differences in ecological and environmental conditions, such as soil composition, altitude, climate, and associated host vegetation, all of which are known to influence secondary metabolite biosynthesis in mushrooms. In particular, abiotic stress factors can stimulate the production of phenolic compounds as part of the fungal defense mechanism against oxidative stress. Moreover, geographic origin has been reported as a critical determinant of antioxidant potential in wild edible mushrooms, with localized environmental pressures shaping species-specific metabolic profiles. The elevated phenolic content observed in *R. roseolus* from Niğde may therefore reflect adaptive metabolic responses unique to this region. These findings highlight the importance of considering geographic and ecological factors when evaluating the bioactive properties of wild mushrooms and support the potential of Niğde-origin *R. roseolus* as a valuable natural source of antioxidant compounds for functional food and pharmaceutical applications.

Kıvrık et al. [35] evaluated eight edible macrofungi, including *R. roseolus*, and demonstrated that antioxidant outcomes depend not only on species but also on the assay type and processing conditions. In their study, ABTS values were generally higher in fresh samples, whereas DPPH (ethanolic) and FRAP (methanolic) activities increased after drying. In line with these observations, the slightly elevated total phenolic content (TPC) detected in the dried *R. roseolus* sample analyzed in the present study, compared with earlier reports from Türkiye, suggests that the Niğde population may be relatively enriched in phenolic compounds. This finding supports the notion that environmental and ecological factors can significantly influence phenolic metabolism in this species [35,36]. Although the strong antioxidant potential of *R. roseolus*, particularly based on IC_50_ values, has been previously reported, the results obtained here indicate that the antioxidant capacity of the Niğde ecotype may be considerably higher than that of samples examined in earlier studies [7,36]. Notably, its elevated DPPH radical scavenging activity compared with many other wild edible mushrooms highlights the potential of this specific ecotype as a promising natural antioxidant source. These characteristics suggest that Niğde-origin *R. roseolus* merits further investigation for possible applications in pharmaceutical and nutraceutical industries.

The relationship between total phenolic content and antioxidant performance observed in this study is consistent with reports emphasizing the central role of phenolic compounds in mushroom antioxidant defense systems [10,37]. Furthermore, the strong reducing power detected in FRAP and CUPRAC assays indicates the presence of effective electron-donating molecules within the extracts. Interestingly, despite a moderate overall TPC, the extracts exhibited pronounced radical scavenging activity, suggesting that phenolic composition and molecular structure, rather than total quantity alone, may be the primary determinants of antioxidant efficiency in *R. roseolus.*

Compared with other members of the *Rhizopogon* genus, the results obtained for *R. roseolus* in the present study further confirm the high bioactive potential characteristic of this genus. *Rhizopogon luteolus*, for instance, has been widely reported to exhibit strong antioxidant activity, rendering it effective not only as an antioxidant but also as an antibacterial agent. Extracts of *R. luteolus* are known to be rich in phenolic acid derivatives, fatty acids, and polysaccharides, and have demonstrated pronounced antioxidant, anti-inflammatory, and antimicrobial effects in vitro, as well as the ability to reduce oxidative stress under experimental sepsis conditions [36]. Such biochemical features suggest that *R. roseolus* may share comparable metabolite profiles and underlying antioxidant mechanisms. The findings of the present study are in agreement with those reported by Sevindik and Bal [38], who, based on TAS (Total Antioxidant Status)/TOS (Total Oxidant Status)/OSI (Oxidative Stress Index) analyses, showed that *R. roseolus* exhibits a more balanced redox status than *R. luteolus*. Although different antioxidant assays were employed, both studies converge on the conclusion that *R. roseolus* possesses a highly efficient antioxidant system. In the current work, the antioxidant capacity of *R. roseolus* was found to be comparable to that reported for *R. luteolus*, reinforcing the notion that these species display similar levels of bioactivity within the genus. In addition, ethnomycological studies on *Rhizopogon vulgaris* indicate that this species is traditionally consumed raw or cooked by local populations, and that bioactive compounds such as ergosterol contribute to its antioxidant properties [11]. Although direct experimental data on the antioxidant capacity of *R. vulgaris* remain limited, available evidence suggests that it contains health-promoting compounds comparable to those identified in *R. roseolus* and *R. luteolus*. Collectively, these observations highlight the *Rhizopogon* genus as a promising source of bioactive metabolites with significant antioxidants and potential therapeutic value. Akata et al. [7] reported that *R. roseolus* extract exhibits high antioxidant activity even at low concentrations. This suggests that the activity of phenolic compounds in *R. roseolus* may be more decisive than their total quantity. In addition to phenolics, other antioxidant constituents commonly found in mushrooms, such as terpenoids, carotenoids, and ascorbic acid, are also likely to contribute to the overall antioxidant capacity. The strong performance of *R. roseolus* in reducing power assays such as CUPRAC and FRAP indicates that this species contains compounds with high reducing potential. In the context of the present study, the notable phenolic content detected in *R. roseolus* may play a central role in its observed antioxidant potential. Therefore, *R. roseolus* can be regarded as a promising natural source of antioxidants.

The bioactive content of mushrooms differs widely depending on species as well as environmental and substrate-related factors. As a result, variations are often observed between studies. Phenolic compounds, including gallic acid, protocatechuic acid, catechin, caffeine, caffeic acid, hydroxybenzoic acid, vanillic acid, chlorogenic acid, and coumaric acid, were identified in certain mushroom species [39,40,41]. Gallic acid, known as an important antioxidant constituent in mushrooms, was detected at a concentration of 552.3 µg/g in the methanolic extract of *Elaphomyces mutabilis* [42]. Gallic acid have been reported in antioxidant-rich mushrooms such as *Ganoderma lucidum* and *Pleurotus ostreatus*, where the high radical scavenging capacity has been largely attributed to their gallic acid content [43,44]. These findings emphasize the diversity in phenolic profiles among mushroom species and their distinct contributions to antioxidant potential. In a previous LC–MS based chemical profiling study, *R. luteolus* was reported to contain acetohydroxamic acid, fumaric acid, salicylic acid, ellagic acid, luteolin, and curcumin, whereas *R. roseolus* was found to include acetohydroxamic acid, fumaric acid, salicylic acid, phloridzin dihydrate, and luteolin [38]. In a previous study on *R. luteolus*, fumaric acid (102 ± 0.002 μg/g), gallic acid (1.50 ± 0.002 μg/g), and protocatechuic acid (1.15 ± 0.001 μg/g) were reported as the major detected compounds [45]. In the present study, a distinct but related phenolic profile was observed, revealing additional phenolic constituents. These differences may be attributed to variations in extraction solvent, analytical approach, or ecological factors influencing metabolite composition.

Fatty acids are essential to metabolic activities such as energy storage and transit, insulation against mechanical, thermal, and electrical influences, signal conveyance and gene regulation, and use as components of cell membranes [46]. The results obtained from this study are in accordance with the previous studies conducted with *R. roseolus* in terms of the predominance of unsaturated fatty acids in the fatty acid composition of the macrofungus. The study investigated the fatty acid composition of 11 wild macrofungi (10 of them were edible) collected from Türkiye, including *R. roseolus,* reported that oleic acid (46.68%) and linoleic acid (38.53%) were the predominant fatty acids determined by GC [47]. The authors also reported that their sample contained small quantities of palmitoleic acid, cis-9 myristoleic acid, cis-10 pentadecenoic acid, and cis-9 heptadecenoic acid. In this study, the concentration of palmitoleic acid was also found less with a value of 3.33%. Another study conducted with *R. luteolus* and *R. roseolus* demonstrated that the macrofungi samples constituted mainly unsaturated fatty acids with concentrations 26.16–42.07% for oleic acid and 35.20–50.30% for linoleic acid, respectively [48]. The authors also reported that the total concentration of unsaturated fatty acids was higher than the total concentration of saturated fatty acids for both specimens. It is well documented that unsaturated fatty acids in the human diet are superior to saturated fatty acids in terms of balancing the cholesterol levels and maintaining cardiac health [49,50,51,52,53,54]. Both the results of this study and the previously reported studies indicated that unsaturated fatty acids were predominant for *R. roseolus,* which was associated with the health benefits.

Edible fungi are typically rich in unsaturated fatty acids such as linoleic and oleic acids. Previous studies have suggested that polyunsaturated fatty acids (PUFAs) may contribute to antioxidant capacity through radical scavenging mechanisms and to antimicrobial activity via disruption of microbial membrane integrity. Importantly, the antimicrobial effectiveness of fatty acids has been reported to depend on their chain length and degree of unsaturation. However, it should be noted that in complex fungus extracts these activities likely result from the combined action of multiple bioactive compounds, including phenolics, terpenes, and polysaccharides. Therefore, while the fatty acid profile may partially explain the observed antioxidant and antimicrobial effects, direct cause of this action cannot be established without targeted bioassays using isolated lipid fractions [55,56].

Mushrooms are rich sources of natural antibiotics, and most of their secondary metabolites are capable of combating various bacteria and viruses. With the increasing number of bacteria developing resistance to commercial antibiotics, extracts and derivatives obtained from fungi offer great potential for the development of new drugs. The extracts of macrofungi are known to possess various levels of antimicrobial activity against pathogenic microorganisms [57]. In another study investigating the antibacterial activity of *R. luteolus* extracts prepared with different solvents, the extracts exhibited antibacterial activity against Gram-positive bacteria including *B. subtilis*, *B. licheniformis*, and *S. aureus*. The hexane extract was reported to inhibit the growth of all tested microorganisms, including *E. coli* [58]. It is well known that Gram-negative microorganisms are generally more resistant to antibacterial agents than Gram-positive ones [8,59]. The antimicrobial activities of *R. luteolus* and *R. roseolus* have been compared, and the results indicated that *R. luteolus* was more effective against bacterial and fungal strains than *R. roseolus* [38]. In another study conducted with the methanolic extract of *R. roseolus* collected from Konya (Türkiye), antibacterial activity was detected against *Citrobacter freundii*, *Enterococcus faecalis*, and *Sarcina lutea* strains at concentrations of 1.56, 0.78, and 0.39 mg/mL, respectively, using the microdilution method. The most effective inhibition was observed against *Staphylococcus aureus* at a concentration of 0.19 mg/mL. However, the extract showed no antifungal activity in either the disk diffusion or microdilution assays [60]. The extracts of *R. luteolus* (RLE) were tested against standard culture collections of the most frequently isolated strains from community- and hospital-acquired infections, following the CLSI recommendations, using the agar dilution method at concentrations ranging from 25 to 800 μg/mL. The antibacterial and antifungal effects of RLE were observed at concentrations between 100 and 400 μg/mL [37]. These findings confirm that the solubility of bioactive components of any macrofungus may vary depending on the extraction solvents used.

Many studies on macrofungi have shown that the efficiency of phenolic extraction and consequent antimicrobial activity strongly depends on the solvent’s polarity. In mushrooms, phenolic compounds with antimicrobial potential are often moderately polar or hydrophobic, and these are generally extracted more effectively with organic solvents such as ethanol or methanol rather than pure water. As a result, ethanolic or methanolic extracts tend to contain higher concentrations of bioactive phenolics and display stronger antimicrobial effects in vitro compared to aqueous extracts, which often yield a narrower phenolic profile and lower antimicrobial efficacy in disk diffusion or MIC assays. This phenomenon has been observed in studies comparing extraction solvents for mushroom bioactives, where the type of solvent significantly influenced total phenolic content and biological activities, with organic solvent extracts typically outperforming water alone in antimicrobial evaluations [42,61,62,63]. Although the highest antimicrobial and antioxidant performance was determined in the methanol extract in all assays, according to the results of this study, both ethanol and water can also be considered adequate solvents for *R. roseolus*.

The variations observed in the antimicrobial activity of this mushroom against the tested microorganisms may be attributed to differences in environmental growth conditions, as factors such as climate, habitat, and soil composition can significantly influence the chemical profile of mushrooms. Although further research is required to elucidate and characterize the bioactive constituents responsible for these effects, the findings presented here provide valuable insights and may serve as a useful reference for future studies focusing on the antimicrobial potential of this macrofungus A major limitation of the present study is the absence of cytotoxicity and biological safety assessments. Therefore, although the extracts showed promising bioactive properties, their potential food or pharmaceutical applications should be interpreted with caution.

## 5. Conclusions

This study represents the first comprehensive assessment of the biological activities associated with methanol, ethanol, and aqueous extracts of *R. roseolus*. The total phenolic content and antioxidant capacity of the extracts were analyzed using four different methods. A GC-MS method was also used to accurately determine the fatty acid composition of *R. roseolus*. It is considered that the high content of phenolic compounds may be responsible for the observed antioxidant effects. The findings provide valuable contributions to the literature on the chemical and biological properties of *R. roseolus* and indicate that this species may be a promising source of natural antioxidant compounds. Furthermore, the antimicrobial activity of *R. roseolus* extracts against various pathogenic microorganisms suggests that this fungus has not only antioxidant capability but also microbial growth inhibitory potential. The fatty acid profile determined by GC-MS analysis showed that it is rich in unsaturated fatty acids, especially oleic and linoleic acid. In addition, the phenolic composition determined by HPLC-DAD revealing the presence of gallic acid, pyrocatechol, and catechin. The present findings demonstrate that this economically important wild edible fungus is source of fatty acids and phenolic compounds, contributing to a better understanding of the biochemical structure of *R. roseolus* and highlighting its potential for future pharmacological and nutraceutical applications. Future studies integrating advanced phytochemical characterization and toxicological analyses are essential to validate safety and support the translation of fungus-derived compounds into functional products.

## Figures and Tables

**Table 1:** Total content of phenolic compounds and antioxidant capacity of *R. roseolus* extracts.

	Extraction Yield (%)	TPC	DPPH	ABTS	FRAP	CUPRAC
MeOH	20.01 ± 0.35c	9.89 ± 0.10c	651.44 ± 15.02b	162.71 ± 8.11b	724.33 ± 12.23b	952.45 ± 4.35b
EtOH	16.19 ± 0.49b	8.97 ± 0.07b	601.01 ± 10.18b	157.68 ± 7.08b	711.52 ± 3.74b	912.49 ± 4.34b
Aq	12.27 ± 0.90a	7.90 ± 0.22a	486.81 ± 12.21a	119.98 ± 2.26a	650.72 ± 5.11a	770.11 ± 31.02a

TPC: Total phenolic compounds, mg GAE/g DW; DPPH: DPPH scavenging activity, mg TE/g DW; ABTS: ABTS scavenging activity, mg TE/g DW; FRAP: Ferric reducing antioxidant power, mg AAE/g DW; CUPRAC: Cupric ion reducing antioxidant capacity, mg TE/g DW; GAE: Gallic acid equivalent; TE: Trolox equivalent; AAE: Ascorbic acid equivalent; DW: Dry weight. The lowercase letters in each column indicate a significant difference at *p* < 0.05.

**Table 2:** Phenolic composition of *R. roseolus* extracts determined by HPLC-DAD.

	RT (min)	R^2^	(µg/g)		
			MeOH	EtOH	Aq
**Gallic acid**	8.442	0.9995	88.83 ± 3.21c	75.24 ± 2.41b	65.02 ± 2.55a
**Pyrocatechol**	14.474	0.9999	169.41 ± 4.24c	120.47 ± 3.47b	79.71 ± 2.78a
**Catechin**	20.701	0.9997	73.24 ± 2.12c	50.69 ± 1.28b	29.48 ± 1.14a
**Caffeic acid**	26.737	0.9995	17.12 ± 0.71b	14.35 ± 0.85ab	12.06 ± 0.92a
**Epicatechin**	27.842	0.9997	33.82 ± 0.45c	26.74 ± 0.74b	20.96 ± 0.67a
**p-coumaric acid**	32.983	0.9987	4.91 ± 0.08b	3.21 ± 0.13ab	2.55 ± 0.09a
**Quercetin**	46.395	0.9988	5.13 ± 0.02b	4.49 ± 0.04ab	3.82 ± 0.01a

RT: retention time (min), R^2^: coefficient of determination, Different lowercase letters in each row indicate a significant difference at *p* < 0.05.

**Table 3:** Fatty acid composition of *R. roseolus* determined by GC-MS.

No.	Compound Name	RT (min)	Mass + Formula	Conc. (%)
1	Myristic acid, methyl ester	32.95	242 (C_15_H_30_O_2_)	0.19
2	Palmitoleic acid, methyl ester	37.842	268 (C_17_H_3_2O_2_)	3.33
3	Palmitic acid, methyl ester	38.098	270 (C_17_H_34_O_2_)	6.42
4	Palmitoleic acid, ethyl ester	39.4	282 (C_18_H_34_O_2_)	0.89
5	Palmitic acid, ethyl ester	39.634	284 (C_18_H_36_O_2_)	1.46
6	Linoleic acid, methyl ester	41.809	294 (C_19_H_34_O_2_)	25.03
7	Oleic acid, methyl ester	41.929	296 (C_19_H_36_O_2_)	35.37
8	Stearic acid, methyl ester	42.429	298 (C_19_H_38_O_2_)	3.44
9	Linoleic acid, ethyl ester	43.147	308 (C_20_H_36_O_2_)	6.35
10	Oleic acid, ethyl ester	43.252	310 (C_20_H_38_O_2_)	8.23
11	Stearic acid, ethyl ester	43.743	312 (C_20_H_40_O_2_)	0.89
12	Squalene	44.596	410 (C_30_H_50_)	6.25
13	8,11,14-Docosatrienoic acid, methyl ester	45.678	348 (C_23_H_40_O_2_)	0.29
14	Arachidic acid methyl ester	46.231	326 (C_21_H_42_O_2_)	0.20
			Total Identified	98.34

RT: Retention time (min) Conc: Relative concentration (%).

**Table 4:** Antimicrobial activity of *R. roseolus* extracts (100 mg/mL) assessed by disk diffusion method.

	Inhibition Zones (mm)				
Test Organism	MeOH	EtOH	Aq	CIP	TOB
*Staphylococcus aureus* ATCC 25923	nd	nd	nd	24.00	14.00
*Bacillus cereus* RSKK 863	22.25 ± 0.35b	21.00 ± 0.71b	16.00 ± 1.41a	36.00	26.00
*Enterobacter aerogenes* ATCC 13048	12.23 ± 0.32b	9.13 ± 0.88a	7.75 ± 0.35a	30.00	18.00
*Pseudomonas aeruginosa* DSMZ 50071	12.45 ± 0.42c	10.38 ± 0.53b	8.50 ± 0.35a	28.00	22.00
*Salmonella typhimurium* SL1344	nd	nd	nd	35.00	15.00
*Bacillus subtilis* DSMZ 1971	25.0 ± 0.35b	24.00 ± 0.71b	17.50 ± 1.06a	36.00	26.00
*Shigella flexneri* RSKK 184	nd	nd	nd	25.00	14.00
*Escherichia coli* ATCC 25922	nd	nd	nd	nd	20.00
*Micrococcus luteus* M41	13.13 ± 0.53b	10.88 ± 1.24ab	8.38 ± 0.18a	27.00	nd
*Proteus vulgaris* ATCC 13315	nd	nd	nd	36.00	11.00
*S. epidermidis* DSMZ 20044	11.00 ± 0.35	nd	nd	34.00	20.00
*Candida albicans* ATCC 10231	nd	nd	nd	nd	13.00
*Enterococcus faecium* (FI)	19.00 ± 0.71b	16.25 ± 1.41ab	12.00 ± 1.06a	28.00	15.00
*Salmonella kentucky* (FI)	nd	nd	nd	24.00	16.00
*Salmonella infantis* (FI)	nd	nd	nd	26.00	14.00
*Klebsiella pneumoniae* (FI)	nd	nd	nd	30.00	23.00
*Listeria innocua* (FI)	8.50 ± 1.06a	7.00 ± 0.35a	nd	18.00	15.00
*Streptococcus mutans* (CI)	14.00 ± 1.41b	12.000.71ab	9.00 ± 1.06a	21.00	24.00
*Candida tropicalis* (CI)	nd	nd	nd	nd	nd
*Klebsiella pneumoniae* (CI)	nd	nd	nd	20.00	18.00
*Staphylococcus aureus* (MRSA)	11.50 ± 1.06a	10.00 ± 0.71a	nd	nd	7.00

Inhibition zones as mean ± standard deviations in mm. nd: Not detected. FI: Food isolate, CI: Clinical isolate. CIP: Ciprofloxacin (5 µg), TOB: Tobramycin (10 µg). Different lowercase letters in each row indicate a significant difference at *p* < 0.05.

## Data Availability

The original contributions presented in the study are included in the article material, further inquiries can be directed to the corresponding author.

## Abbreviations

The following abbreviations are used in this manuscript:

ITS	Internal Transcribed Spacer
DPPH	2,2-diphenyl-1-picrylhydrazyl
ABTS	2-azino-bis-(3-ethylbenzothiozoline-6-sulphonic acid)
FRAP	Ferric Reducing Antioxidant Power
CUPRAC	Cupric Reducing Antioxidant Capacity
TPC	Total Phenolic Compounds
HPLC-DAD	High Performance Liquid Chromatography-Diode Array Detection
GC-MS	Gas Chromatography-Mass Spectrometry

## References

[1] Aytar E.C., Özmen A. (2020). Cytotoxic and apoptotic activities of *Rhizopogon roseolus* (Corda) Th. Fr. extracts. Int. J. Second. Metab..

[2] Koizumi T., Nara K. (2016). Two new species of *Rhizopogon* associated with *Pinus pumila* from Japan. Mycoscience.

[3] Çelik A., Uzun Y., Kaya A. (2020). Macrofungal Biodiversity of Güneysınır District (Konya-Turkey). J. Fungus.

[4] Nuñez J.D., Saiz M., Calderon C., de Omeñaca González J.A.S. (2013). Physiological effects of *Rhizopogon roseolus* on *Pinus halepensis* seedlings. For. Syst..

[5] Okuda Y., Shimomura N., Funato C., Nagasawa E., Matsumoto T. (2013). Genetic variation among natural isolates of the ectomycorrhizal hypogenous fungus, *Rhizopogon roseolus* from Japanese pine forests inferred using AFLP markers. Mycoscience.

[6] Łysakowska P., Sobota A., Wirkijowska A. (2023). Medicinal mushrooms: Their bioactive components, nutritional value and application in functional food production—A review. Molecules.

[7] Akata I., Ergonul B., Kalyoncu F. (2012). Chemical compositions and antioxidant activities of 16 wild edible mushroom species grown in Anatolia. Int. J. Pharmacol..

[8] Yamaç M., Bilgili F. (2006). Antimicrobial activities of fruit bodies and/or mycelial cultures of some mushroom isolates. Pharm. Biol..

[9] Solak M.H., Kalmış E., Sağlam H., Kalyoncu F. (2006). Antimicrobial activity of two wild mushrooms *Clitocybe alexandri* (Gill.) Konr. and *Rhizopogon roseolus* (Corda) T.M. Fries collected from Turkey. Phytother. Res..

[10] Kozarski M., Klaus A., Jakovljevic D., Todorovic N., Vunduk J., Petrović P., Nikšić M., Vrvic M.M., Griensven L.V. (2015). Antioxidants of edible mushrooms. Molecules.

[11] Rangsinth P., Sharika R., Pattarachotanant N., Duangjan C., Wongwan C., Sillapachaiyaporn C., Nilkhet S., Wongsirojkul N., Prasansuklab A., Tencomnao T. (2023). Potential beneficial effects and pharmacological properties of ergosterol, a common bioactive compound in edible mushrooms. Foods.

[12] Álvarez-Martínez F.J., Barrajón-Catalán E., Micol V. (2020). Tackling antibiotic resistance with compounds of natural origin: A comprehensive review. Biomedicines.

[13] Gursoy N., Sarikurkcu C., Cengiz M., Solak M.H. (2009). Antioxidant activities, metal contents, total phenolics and flavonoids of seven *Morchella* species. Food Chem. Toxicol..

[14] Sezgin S., Dalar A., Uzun Y. (2020). Determination of antioxidant activities and chemical composition of sequential fractions of five edible mushrooms from Turkey. J. Food Sci. Technol..

[15] Breitenbach J., Kränzlin F. (1991). Fungi of Switzerland, Volume 3, Boletes and Agarics 1. Part.

[16] Hansen L., Knudsen H. (1992). Nordic Macromycetes, Volume 2, Polyporales, Boletales, Agaricales, Russulales.

[17] Jordan M. (2004). The Encyclopedia of Fungi of Britain and Europe.

[18] Kränzlin F. (2005). Fungi of Switzerland, Volume 6, Russulaceae 2.

[19] Lücking R., Aime M.C., Robbertse B., Miller A.N., Ariyawansa H.A., Aoki T., Cardinali G., Crous P.W., Druzhinina I.S., Geiser D.M. (2020). Unambiguous identification of fungi: Where do we stand and how accurate and precise is fungal DNA barcoding?. IMA Fungus.

[20] Giusti A., Ricci E., Gasperetti L., Galgani M., Polidori L., Verdigi F., Narducci R., Armani A. (2020). Molecular identification of mushroom species in Italy: An ongoing project aimed at reinforcing the control measures of an increasingly appreciated sustainable food. Sustainability.

[21] White T.J., Bruns T., Lee S.J.W.T., Taylor J., Innis M.A., Gelfand D.H., Sninsky J.J., White T.J. (1990). Amplification and direct sequencing of fungal ribosomal RNA genes for phylogenetics. PCR Protocols: A Guide to Methods and Applications.

[22] Dostemessova A., Canpolat Ş., Kurmanbayeva M., Sadykova D., Yürümez Canpolat E., Ildız E., Izbastina K., İşlek C. (2025). Biological Activity and Alkaloid Composition of *Chelidonium majus* L. Growing at Three Sites in the Eastern Part of Kungey Alatau Ridge (Kazakhstan). Appl. Biol. Res..

[23] Singleton V.L., Rossi J.A. (1965). Colorimetry of total phenolics with phosphomolybdic- phosphotungstic acid reagents. Am. J. Enol. Vitic..

[24] Silici S., Sarioglu K., Dogan M., Karaman K. (2014). HPLC-DAD analysis to identify the phenolic profile of rhododendron honeys collected from different regions in Turkey. Int. J. Food Prop..

[25] Blois M.S. (1958). Antioxidant determinations by the use of a stable free radical. Nature.

[26] Silva F., Veiga F., Cardoso C., Dias F., Cerqueira F., Medeiros R., Paiva-Santos A.C. (2024). A rapid and simplified DPPH assay for analysis of antioxidant interactions in binary combinations. Microchem. J..

[27] Mohtar L.G., Messina G.A., Bertolino F.A., Pereira S.V., Raba J., Nazareno M.A. (2020). Comparative study of different methodologies for the determination the antioxidant activity of Venezuelan propolis. Microchem. J..

[28] Mokrani A., Krisa S., Cluzet S., Da Costa G., Temsamani H., Renouf E., Mérillon J.M., Madani K., Mesnil M., Monvoisin A. (2016). Phenolic contents and bioactive potential of peach fruit extracts. Food Chem..

[29] Apak R., Güçlü K., Özyürek M., Esin Karademir S., Erçağ E. (2006). The cupric ion reducing antioxidant capacity and polyphenolic content of some herbal teas. Int. J. Food Sci. Nutr..

[30] Canpolat Ş., Canpolat E.Y., İşlek C. (2024). The Biological Activities of *Hypericum perforatum* L.. ISPEC J. Agric. Sci..

[31] Zoral F., Turgay Ö. (2014). A Research on Total Phenolic Content, Antioxidant Activity and Antimicrobial Effects of Various Food Wastes. KSU J. Nat. Sci..

[32] Gursoy N., Bektas T., Halil S.M. (2010). Evaluation of antioxidant activities of three edible mushrooms; *Ramaria flava*, *Rhizopogan roseolus* and *Russula delica*. Food Sci. Biotech..

[33] Tel-Çayan G., Muhammad A., Deveci E., Duru M.E., Öztürk M. (2020). Isolation, structural characterization, and biological activities of galactomannans from *Rhizopogon luteolus* and *Ganoderma adspersum* mushrooms. Int. J. Biol. Macromol..

[34] Bayram O.F., Marah S., Turkekul I., Ozen T. (2024). Phytochemical profile, bioactivity, and molecular docking studies of natural edible mushrooms grown in Tokat and Sivas provinces of Turkey. J. Food Sci..

[35] Kıvrık M., Süfer Ö., Bozok F. (2022). A Research on Quality Evaluation of Eight Wild Edible Macrofungi Collected from East Mediterranean Region of Turkey. Chem. Biodivers..

[36] Ozhan O., Yildiz A., Balcioglu S., Gunal S., Vardi N., Ates B., Gunata M., Parlakpinar H. (2025). In vitro antioxidant and antimicrobial activities and therapeutic effects of *Rhizopogon luteolus* Fr. extract on cecal ligation and punctureinduced sepsis in rats. Discov. Appl. Sci..

[37] Heleno S.A., Ferreira R.C., Antonio A.L., Queiroz M.J.R., Barros L., Ferreira I.C. (2015). Nutritional value, bioactive compounds and antioxidant properties of three edible mushrooms from Poland. Food Biosci..

[38] Sevindik M., Bal C. (2022). Chemical Characterization, Antibacterial, Antifungal, Antioxidant and Oxidant Activities of Wild Mushrooms *Rhizopogon luteolus* and *Rhizopogon roseolus*. Biol. Bull..

[39] Silva M., Lageiro M., Ramos A.C., Reboredo F.H., Gonçalves E.M. (2024). Cultivated mushrooms: A comparative study of antioxidant activity and phenolic content. Biol. Life Sci. Forum.

[40] Fogarasi M., Socaciu M.I., Sălăgean C.D., Ranga F., Fărcaș A.C., Socaci S.A., Socaciu C., Tibulca D., Fogarasi S., Semeniuc C.A. (2021). Comparison of different extraction solvents for characterization of antioxidant potential and polyphenolic composition in *Boletus edulis* and *Cantharellus cibarius* mushrooms from Romania. Molecules.

[41] Çayan F., Tel-Çayan G., Deveci E., Duru M.E. (2021). HPLC–DAD characterization of phenolic profile and in vitro antioxidant, anticholinesterase, and antidiabetic activities of five mushroom species from Turkey. 3 Biotech.

[42] Ulusu F., Emsen B., Karapinar H.S., Uzun Y., Kaya A. (2025). HPLC based identification of phenolics and biological activities in Elaphomyces mushroom extracts. Sci. Rep..

[43] Tel-Çayan G., Muhammad A., Duru M.E., Öztürk M., Adhikari A., Türkoğlu A. (2016). A new fatty acid ester from an edible mushroom *Rhizopogon luteolus*. Nat. Prod. Res..

[44] Atila F., Ogutcu H., Bilginoglu E., Kazankaya A., Kumar P., Fayssal S.A. (2024). Effect of phenolic-rich forest and Agri-food wastes on yield, antioxidant, and antimicrobial activities of *Ganoderma lucidum*. Biomass Convers. Biorefinery.

[45] Gąsecka M., Mleczek M., Siwulski M., Niedzielski P. (2016). Phenolic composition and antioxidant properties of *Pleurotus ostreatus* and *Pleurotus eryngii* enriched with selenium and zinc. Eur. Food Res. Technol..

[46] Rustan A.C., Drevon C.A. (2005). Fatty acids: Structures and properties. Encycl. Life Sci..

[47] Zengin G., Sarikurkcu C., Aktumsek A., Uysal S., Ceylan R., Anwar F., Solak M.H. (2015). A Comparative fatty acid compositional analysis of different wild species of mushrooms from Turkey. Emir. J. Food Agric..

[48] Işık H., Bengü A.Ş., Yılmaz H.Ç., Yılmaz N., Türkekul İ. (2020). A study on mineral contents and fatty acid profiling of two *Rhizopogon* species. Acta Biol. Turc..

[49] Caggiula A.W., Mustad V.A. (1997). Effects of dietary fat and fatty acids on coronary artery disease risk and total and lipoprotein cholesterol concentrations: Epidemiologic studies. Am. J. Clin. Nutr..

[50] Jakobsen M.U., O’Reilly E.J., Heitmann B.L., Pereira M.A., Bälter K., Fraser G.E., Goldbourt U., Hallmans G., Knekt P., Liu S. (2009). Major types of dietary fat and risk of coronary heart disease: A pooled analysis of 11 cohort studies. Am. J. Clin. Nutr..

[51] Skeaff C.M., Miller J. (2009). Dietary fat and coronary heart disease: Summary of evidence from prospective cohort and randomised controlled trials. Ann. Nutr. Metab..

[52] Salter A.M. (2013). Dietary fatty acids and cardiovascular disease. Animal.

[53] Hammad S., Pu S., Jones P.J. (2016). Current evidence supporting the link between dietary fatty acids and cardiovascular disease. Lipids.

[54] Mazur M., Przytuła A., Szymańska M., Popiołek-Kalisz J. (2024). Dietary strategies for cardiovascular disease risk factors prevention. Curr. Probl. Cardiol..

[55] Tan R.Y., Ilham Z., Wan W.A.A.Q.I., Halim-Lim S.A., Usuldin S.R.A., Ahmad R., Adlim M. (2024). Mushroom oils: A review of their production, composition, and potential applications. Heliyon.

[56] Chanda W., Joseph T.P., Guo X.F., Wang W.D., Liu M., Vuai M.S., Padhiar A.A., Zhong M.T. (2018). Effectiveness of omega-3 polyunsaturated fatty acids against microbial pathogens. J. Zhejiang Univ. Sci. B.

[57] Barros L., Calhelha R.C., Vaz J.A., Ferreira I.C., Baptista P., Estevinho L.M. (2007). Antimicrobial activity and bioactive compounds of Portuguese wild edible mushrooms methanolic extracts. Eur. Food Res. Technol..

[58] Sadi G., Emsen B., Kaya A., Kocabaş A., Çınar S., Kartal D.İ. (2015). Cytotoxicity of some edible mushrooms extracts over liver hepatocellular carcinoma cells in conjunction with their antioxidant and antibacterial properties. Phcog. Mag..

[59] Ranadive K.R., Belsare M.H., Deokule S.S., Jagtap N.V., Jadhav H.K., Vaidya J.G. (2013). Glimpses of antimicrobial activity of fungi from World. J. New Biol. Rep..

[60] Yaprak A., Alkan S., Güneş E., Kaşık G. (2024). Investigation of the Antimicrobial Activities of Some Macrofungi Growing in the Kestel Region (Kadınhanı-Konya). Karaelmas Sci. Eng. J..

[61] Kim J.H., Tam C.C., Chan K.L., Mahoney N., Cheng L.W., Friedman M., Land K.M. (2022). Antimicrobial efficacy of edible mushroom extracts: Assessment of fungal resistance. Appl. Sci..

[62] Ewunkem A., Merrills L., Williams Z., Justice B., Iloghalu U., Williams V., Singh D. (2024). In Vitro Antimicrobial Efficacy Assessment of Ethanolic, Aqueous, and Dual Solvent Extracts of Mushroom *Ganoderma lucidum*: Genomic and Morphological Analysis. Antibiotics.

[63] Michalska A., Sierocka M., Drzewiecka B., Świeca M. (2025). Antioxidant and Anti-Inflammatory Properties of Mushroom-Based Food Additives and Food Fortified with Them—Current Status and Future Perspectives. Antioxidants.

